# The Multi-Target lncRNA–miRNA–mRNA TRIAD in Pancreatic Cancer Diagnosis and Therapy

**DOI:** 10.3390/ijms27031400

**Published:** 2026-01-30

**Authors:** Hyeon-su Jeong, Yun Ju Lee, Du Hyeong Lee, Hyun-Young Roh, Ga-ram Jeong, Heui-Soo Kim

**Affiliations:** 1 Department of Integrated Biological Science, Pusan National University, Busan 46241, Republic of Korea; 2 Institute of Systems Biology, Pusan National University, Busan 46241, Republic of Korea; 3 Department of Biological Sciences, College of Natural Sciences, Pusan National University, Busan 46241, Republic of Korea

**Keywords:** pancreatic cancer, protein-coding gene, non-coding RNA, miRNA, lncRNA, TRIAD, ceRNA, muti-target, treatment, biomarker

## Abstract

Pancreatic cancer (PC) is one of the most lethal malignancies worldwide, characterized by late diagnosis, aggressive progression, and limited responsiveness to current therapeutic strategies. Although extensive genomic analyses have identified key driver protein-coding genes (PCGs), therapeutic approaches targeting individual genes have shown limited clinical benefit. This limitation highlights the molecular complexity of PC, where tumor progression is governed by regulatory networks that extend beyond genetic alterations. Non-coding RNAs (ncRNAs), which constitute nearly 98% of the human genome, have emerged as regulators of gene expression in cancer. Among them, microRNAs (miRNAs) and long non-coding RNAs (lncRNAs) regulate oncogenic processes, including aberrant signaling activation, tumor microenvironment remodeling, epithelial–mesenchymal transition, immune evasion, and resistance. Beyond their independent functions, lncRNAs, miRNAs, and mRNAs form an integrated regulatory network known as the lncRNA–miRNA–mRNA TRIAD, enabling control of gene expression. Such network-based regulation provides a framework for multi-target therapeutic strategies. Moreover, the rapid responsiveness and disease-specific expression patterns of ncRNAs suggest strong potential as diagnostic and prognostic biomarkers in PC, where early detection remains challenging. This review summarizes the regulatory roles of PCGs, miRNAs, and lncRNAs in PC and highlights the lncRNA–miRNA–mRNA TRIAD as a framework for understanding gene regulatory networks.

## 1. Introduction

Pancreatic cancer (PC) is one of the most lethal malignancies worldwide, with the incidence increasing substantially from approximately 207,905 to 508,333 new cases [1]. The deep anatomical location of the pancreas permits early-stage tumors to progress without overt symptoms, rendering PC difficult to detect and treat at an early stage [2]. So, despite advances in cancer research and treatment, the global burden of PC has more than doubled over recent decades, with deaths rising from approximately 211,613 in 1990 to 467,409 in 2022 [3]. Among major cancers, PC exhibits the lowest five-year survival rate, which remains at approximately 12% [4]. This poor survival outcome underscores the limited effectiveness of currently available therapeutic strategies and highlights the urgent need for more effective treatment approaches [5].

PC develops through the abnormal proliferation of pancreatic cells that lose normal regulatory control [6]. PC progression is influenced by complex interactions among genetic, environmental, and lifestyle factors, with a dominant contribution from genetic factors [7]. PC is generally associated with variants in four genes, *KRAS*, *TP53*, *CDKN2A*, and *SMAD4*, which are widely regarded as the main driver genes of PC [8,9,10,11,12]. These genes contribute to carcinogenesis by enhancing cellular proliferation, metabolic reprogramming, and immune evasion, and are also implicated in resistance to chemotherapy and radiotherapy [13,14]. Beyond these core driver alterations, the progressive accumulation of additional genomic abnormalities further disrupts key biological processes, including signaling pathways and the TME, thereby exacerbating PC development and progression [15]. The accumulation of diverse genomic abnormalities and alterations has been shown to affect various biological processes, including signaling pathways and the tumor microenvironment (TME), further driving PC development [16]. In this context, numerous studies have concentrated on therapeutic strategies that target individual molecular elements, including major driver genes, as single targets [17]. However, unlike in several other cancers, such single-target approaches have shown limited therapeutic efficacy in PC [18,19]. This limited response is because single-target treatments are considered insufficient to address the complex molecular characteristics [20]. Therefore, multi-target therapeutic approaches are needed, making the identification of appropriate targets a critical step in effective strategy development [21].

With the continued expansion of related research, increasing attention has been directed toward non-coding RNAs (ncRNAs), which constitute nearly 98% of the human genome and serve as key regulators of gene expression [22]. Unlike protein-coding transcripts, ncRNAs are not translated into proteins but instead function as regulatory molecules in their RNA form [23]. These ncRNAs have emerged as critical modulators of hallmark cancer phenotypes, including uncontrolled proliferation, invasion, and metastasis [24]. In PC, dysregulated ncRNA expression has been widely reported to promote tumor initiation and progression [25]. Among the major ncRNA subclasses, long non-coding RNAs (lncRNAs) and microRNAs (miRNAs) interact with mRNAs to form dynamic regulatory networks known as competing endogenous RNA (ceRNA) networks. In this context, the TRIAD refers to the three core regulatory components, lncRNAs, miRNAs, and mRNAs, that constitute the ceRNA system [26,27]. These lncRNA–miRNA–mRNA interactions enable control of gene expression [28]. Such network-based regulation may partially overcome the limitations of single-target therapeutic strategies, which have shown limited efficacy in PC [29,30]. Accordingly, the following sections discuss the biological functions and significance of individual ncRNA subclasses, as well as their integrated regulatory roles within the lncRNA–miRNA–mRNA TRIAD.

Moreover, TRIAD incorporates ncRNAs that exhibit rapid responses to pathological changes, suggesting potential value as biomarkers in PC, where early detection remains challenging [31]. A comprehensive understanding of the fundamental biological functions and interactions of lncRNAs, miRNAs, and mRNAs is consequently essential. In this review, we aim to identify TRIADs that may serve as key therapeutic targets in PC, thereby highlighting potential candidates for multi-target therapeutic strategies and diagnosis.

## 2. Regulation of Protein-Coding Genes (PCGs) in PC

Protein-coding genes (PCGs) constitute only about 2% of the human genome, but they are essential for cellular physiology as they encode the information required to produce functional proteins [32]. A wide range of PCGs are expressed in the human body, and their transcripts, messenger RNAs (mRNAs), regulate diverse cellular processes [33,34]. Cancer, the leading cause of mortality worldwide, arises from multiple factors, but is ultimately driven by dysregulated expression of specific PCGs [35,36,37]. PCGs include oncogenes, which promote malignant transformation, and tumor suppressor genes (TSGs) that restrain tumor development [38]. Dysregulation of PCGs, either through overexpression of oncogenes or suppression of TSGs, facilitates tumorigenesis [39,40].

PC also develops as a result of dysregulation of PCGs involved in processes such as cell growth, metabolism, and immune evasion [41,42]. In other words, these PCG expression changes contribute significantly to PC progression [43,44]. Although all these dysregulated PCGs ultimately drive PC progression, differences exist in the underlying mechanisms of carcinogenesis (Table 1). Therefore, in this section, we focus on the representative carcinogenic mechanisms associated with alterations in the expression of PCGs and discuss how these factors influence PC.

### 2.1. Regulation of PCGs Associated with Signaling Pathways

In the development of PC, PCGs are most frequently implicated through their involvement in diverse intracellular signaling pathways [65,66]. In the normal pancreas, oncogenes and TSGs maintain an antagonistic balance that precisely regulates cell growth, division, survival, metabolism, and stress responses [67,68]. This tightly controlled regulatory network ensures appropriate cellular responses to physiological signals and preserves pancreatic homeostasis [55]. However, oncogene overactivation or loss of TSG function disrupts intracellular signaling balance, causing pancreatic cells to fail to respond properly to regulatory cues [69,70]. As a result, these cells constitutively activate proliferation and survival pathways, thereby contributing to abnormal pancreatic cell physiology, malignant transformation, and ultimately pancreatic tumorigenesis [70].

In PC, oncogenes primarily function within growth factor signaling pathways, typically inducing abnormal activation of the Mitogen-Activated Protein Kinase (MAPK) signaling pathway, Wnt signaling pathway, PI3K–AKT–mTOR signaling pathway, and JAK–STAT signaling pathway [71]. For example, overexpression of *KRAS*, which functions as an oncogene in PC, maintained persistent ERK signaling in the MAPK signaling pathway even in the absence of upstream ligand stimulation [45]. Consequently, sustained signaling promoted the transcription of cell cycle-related genes in cancer cells, ultimately driving tumorigenesis. Overexpression of another oncogene, *MYC*, was demonstrated to increase the expression of LEF1, which promoted nuclear retention of β-catenin. The resulting increase in nuclear β-catenin activated WNT/β-catenin signaling and thereby enhanced tumor cell proliferation and survival, contributing to PC development [46]. In addition, upregulation of the *LETM2* oncogene increased phosphorylation of PI3K and AKT without altering total protein levels. As a result, the PI3K–AKT signaling pathway was activated, resulting in increased tumor cell proliferation, migration, and invasion, and decreased apoptosis, thereby accelerating PC progression [47]. With respect to *HIST3H2A*, enrichment analyses indicated that *HIST3H2A* enhanced the JAK–STAT signaling pathway. Enhanced JAK–STAT signaling was associated with suppression of antitumor immune responses and promotion of tumor cell growth, thereby contributing to PC development and poor prognosis [49]. Furthermore, aberrant activation of oncogenes was observed across multiple pathways, including signaling pathways beyond growth factor–mediated cascades, such as the Notch signaling pathway, the BMP/SMAD signaling pathway, and metabolism-related signaling networks, thereby further promoting malignant cellular activity.

In contrast, TSGs function to regulate these signaling pathways in order to maintain or restore cellular homeostasis [71]. For instance, *HNF1A* suppresses the PI3K–AKT signaling pathway, inducing cell cycle arrest and apoptosis in PC cells, thereby inhibiting tumor cell growth [55]. *SOX15* suppresses the Wnt/β-catenin signaling pathway by downregulating β-catenin–dependent transcriptional activity, leading to reduced expression of Wnt target genes (TGs) such as *MYC*, *CCND1*, and *LEF1*. Through this inhibition, *SOX15* induces decreased cell proliferation and tumor growth in PC cells [56]. Another TSG, *SMAD4*, facilitated direction of TGF-β signaling toward the TGF-β–SMAD2/3–SMAD4 pathway in normal cells, resulting in tumor-suppressive transcriptional programs and suppression of PC progression. In cancer cells, the absence of *SMAD4* did not block TGF-β signaling but instead redirected it toward non-canonical, *SMAD4*-independent pathways, thereby promoting PC progression through TME remodeling, enhanced metastasis, and poor prognosis [57]. In contrast to the oncogene *LETM2*, which activated the PI3K–AKT signaling pathway, the TSG, *PTEN*, inhibited AKT membrane binding and phosphorylation by extinguishing PI3K-generated signals through dephosphorylation of PIP3 to PIP2 on the plasma membrane. Such regulation prevented excessive activation of pathways regulating cell proliferation, survival, and metabolic reprogramming. That is, *PTEN* functioned as a key regulator of PI3K–AKT pathway homeostasis under physiological conditions, restraining pancreatic epithelial cells from aberrant responses to stress or growth signals [58]. As demonstrated by this example, oncogenes and TSGs did not always act independently and formed dynamic mutual inhibitory relationships within the same signaling axis [72]. This opposing regulation within a shared signaling pathway exemplifies how oncogenes and TSGs exert counterbalancing effects on the same molecular circuits rather than functioning in isolation [73]. Unlike the balanced signaling state maintained in normal cells, the disruption of this antagonistic regulation in cancer results in persistent activation of oncogenic pathways, thereby driving oncogenesis [68].

Taken together, aberrant activation of oncogenes and loss of TSG function disrupt key intracellular signaling networks in PC, resulting in sustained proliferative and survival signaling and enhanced invasive and metastatic potential [74]. This imbalance promotes autonomous cancer cell growth through coordinated effects on cell cycle regulation, metabolic reprogramming, and immune modulation [75]. Consequently, PCGs act as central regulators of PC pathophysiology and represent important therapeutic targets and prognostic biomarkers.

### 2.2. Regulation of PCGs Associated with TME Remodeling

PCGs contribute not only to the regulation of intracellular signaling pathways but also to the remodeling of the TME [75]. These alterations promote a pro-tumorigenic environment through angiogenesis, immune suppression, extracellular matrix (ECM) remodeling, and epithelial–mesenchymal transition (EMT) [76,77,78]. TME comprises a complex and dynamic ecosystem that supports tumor growth and progression [79,80,81,82]. For example, the activation of key oncogenes, such as *YAP1*, has been demonstrated to induce the secretion of various growth factors and cytokines, including VEGF and TGF-β, thereby promoting angiogenesis [50]. This activation also stimulated cancer-associated fibroblasts, which in turn enhanced the infiltration of immunosuppressive cells, ultimately remodeling the TME. Oncogenes such as *MET* and *DCLK1* induce EMT, enabling cancer cells to acquire enhanced motility and invasiveness [51]. Following EMT, the expression of matrix metalloproteinases and other ECM-degrading enzymes was upregulated, thereby reprogramming the structure of the ECM. The remodeling of the ECM and the increased secretion of EMT-associated cytokines promoted the infiltration of immunosuppressive cells, thereby contributing to the formation of a pro-tumorigenic microenvironment. Moreover, the remodeling of the ECM induced by oncogenes led to structural changes in the extracellular matrix that facilitated tumor invasion and metastasis [83,84]. *TWIST1*, a key EMT regulator, directly enhanced the motility and invasiveness of cancer cells by suppressing E-cadherin and upregulating mesenchymal markers [53]. In addition, *TWIST1* not only regulated the network of EMT-related genes but also modulated the expression of the immune checkpoint molecule V-domain Ig-containing suppressor of T cell activation, thereby contributing to the establishment of an immunosuppressive tumor microenvironment. These findings demonstrated that EMT is not merely a morphological change but is functionally linked to the immunosuppressive system [85].

TSGs suppress aberrant cancer cell proliferation and limit imbalances within the TME by regulating immune cell activity and cellular metabolism. For example, *ARID1A* modulates CD8+ T cell infiltration and stromal fibrosis, thereby promoting a TME that induces immune cell penetration. The maintenance of immune cell homeostasis within the TME suppresses PC cell development and carcinogenesis by sustaining antitumor immune responses [60]. Similarly, *PCDH17* regulates the state of endothelial cells, thereby enhancing overall T cell infiltration [61]. Moreover, by sustaining the expression of chemokines involved in T cell recruitment, its loss promotes pro-tumorigenic remodeling of the TME, contributing to tumorigenesis. Additionally, *CEBPA* binds to the cell cycle-promoting transcription factor E2F1, leading to cell cycle arrest and growth inhibition [62]. Through this interaction, *CEBPA* indirectly regulates ECM remodeling and immune responses, thereby suppressing PC progression. Furthermore, certain TSGs regulate the expression of immunosuppressive factors or maintain cell adhesion and polarity, thereby restricting tumor cell invasion and metastasis [86,87]. The functional maintenance of these TSGs stabilizes inflammatory, metabolic, and immune balances within the TME, which are a critical determinant in preventing tumor development [87].

## 3. Regulation of microRNAs (miRNAs) in PC

ncRNAs, which do not encode proteins, were once regarded as non-functional “junk” genes [88]. However, research into ncRNAs has continued due to the fact that ncRNAs constitute an overwhelming majority of the human genome, accounting for approximately 98%, thereby revealing that ncRNAs contain elements involved in transcriptional regulation [89,90]. Recent studies have revealed that ncRNAs perform diverse functions as RNA molecules themselves and are classified into small ncRNAs and lncRNAs based on their length [91]. miRNA, a representative small ncRNA, was first identified in *Caenorhabditis elegans* by Victor Ambros in 1993, while concurrently, the first TG of miRNA was discovered by Gary Ruvkun [92]. Through continued investigation of miRNAs, their role as post-transcriptional regulators of gene expression was established [93]. These miRNAs have been confirmed to function beyond the regulation of individual mRNA targets, coordinating gene regulatory networks that influence diverse physiological processes, including cell growth, metabolism, and immune regulation [94,95]. Recent studies revealed that the dysregulation of miRNAs plays a significant role in various diseases, including cancer, cardiovascular disease, and neurodegenerative diseases [96,97,98]. Furthermore, owing to their rapid expression changes during early disease stages, miRNAs have emerged as promising early diagnostic biomarkers for malignancies that are difficult to detect at an early stage, including PC [96,99]. Therefore, in this section, we focus on representative miRNAs implicated in PC and discuss the oncogenic mechanisms through which they influence disease progression, as well as their potential as therapeutic targets and biomarkers.

### 3.1. miRNA Regulations Associated with the Target Genes (TGs)

miRNAs are small ncRNAs of 18–25 nucleotides that are produced through the nuclear and cytoplasmic processing of primary miRNA transcripts that are transcribed from miRNA genes [100]. miRNAs are loaded onto argonaute proteins to form the RNA-induced silencing complex, after which mature miRNAs bind to complementary miRNA response elements (MREs) within the 3′ untranslated regions (UTRs) of target mRNAs via their seed regions [101]. Complete complementarity with the seed region leads to mRNA degradation, while partial complementarity inhibits mRNA translation [101]. Recent advances in miRNA research have substantially improved our understanding of the mechanisms that regulate gene expression.

miRNA-mediated regulation has been shown to influence a wide range of biological processes in cancer cells, including cell cycle progression, differentiation, apoptosis, metabolism, inflammation, and DNA damage responses. In normal cells, miRNAs play a critical role in maintaining gene expression homeostasis. In contrast, in cancer cells, miRNA expression is profoundly dysregulated, leading to disruption of cellular homeostasis and contributing to tumor development. Consequently, miRNAs are functionally classified as oncogenic miRNAs (oncomiRs) or tumor-suppressive miRNAs (TSmiRs) according to their overall effects on cancer-related gene regulation.

For example, miR-155 binds to the TG *FoxO3a* to induce ROS generation and suppresses antioxidants such as SOD2 and catalase, thereby promoting proliferation in PC cells [102]. In addition to directly suppressing apoptosis-related genes or TSGs, miRNAs can indirectly modulate oncogenic signaling pathways by regulating TGs that function as key components of these pathways. For instance, miR-642a-5p inhibit the TG *KRT19*, thereby activating the Wnt signaling pathway. Activation of Wnt signaling consequently promotes EMT, PC cell proliferation, migration, and invasion, thereby driving tumorigenesis [103]. Furthermore, miRNAs can also modify the TME to create a tumor-favorable environment. For example, miR-194-5p was shown to directly bind to the 3′ UTR of the target gene *PD-L1*, thereby suppressing *PD-L1* expression. Suppression of *PD-L1* regulates immune cell activity and immune checkpoint-associated signaling pathways, remodels the TME, and enhances tumorigenicity [104].

In contrast, TSmiRs exhibit anti-cancer functions in normal cells but are downregulated in cancer cells, resulting in aberrant expression of oncogenes. For example, miR-217 directly binds to the 3′ UTR of *KRAS*, reducing KRAS protein expression. Reduction in KRAS expression decreases downstream signaling, including p-AKT, thereby suppressing PC cell growth and anchorage-independent colony formation [105]. MiR-30d was shown to target *SOX4* and reduce tumor survival signaling by inhibiting activation of the PI3K–AKT pathway [106]. Overexpression of miR-30d subsequently suppresses proliferation, migration, and invasion of PC cells, induces G1/S cell cycle arrest, and increases apoptosis, thereby inhibiting tumor growth. Additionally, miR-202 directly binds to *HK2*, reducing *HK2* mRNA and protein expression. Suppression of *HK2* inhibits glycolysis, resulting in decreased glucose-6-phosphate levels, glucose uptake, lactate production, and ATP generation, thereby reducing cell proliferation and tumor formation [107]. MiR-128-3p was reported to inhibit EMT by targeting *ZEB1*, leading to increased E-cadherin and decreased N-cadherin expression. Suppression of EMT consequently reduces cell migration and invasion, thereby inhibiting cancer progression [108]. Meanwhile, miR-190b has been reported to suppress cell proliferation by repressing *MEF2C* and *TCF4*. Repression of *MEF2C* and *TCF4* inhibits the invasion–metastasis axis and the Wnt/β-catenin transcriptional axis, thereby reducing proliferation, invasion, and metastasis of PC cells [109].

Overall, miRNAs play central roles in PC progression by post-transcriptionally regulating cancer-related genes, thereby shaping intracellular signaling pathways and interactions with the TME. Depending on their regulatory targets and functional contexts, miRNAs act either as oncogenic drivers or tumor suppressors, highlighting their relevance as key modulators of tumor biology and as potential biomarkers and therapeutic targets in PC (Table 2).

### 3.2. Noncanonical Regulation of miRNAs

As discussed in Section 3.1, miRNAs typically regulate cancer progression by binding to their TGs and modulating post-transcriptional gene expression. However, recent studies have revealed that miRNAs can also exert noncanonical functions, including direct regulation at the transcriptional level and interaction with transcription factors (TFs) or chromatin regulatory complexes [122]. In addition, noncanonical miRNAs contributed to remodeling the TME by influencing immune cell infiltration, stromal activation, and signaling pathway dynamics [122,123]. These effects are similar to those of PCGs and highlight the multifaceted roles of miRNAs beyond their classical post-transcriptional functions.

For example, a distinct subset of noncanonical miRNAs, referred to as nuclear activating miRNAs (NAmiRNAs), has been identified to regulate gene expression at the transcriptional level [123]. NAmiRNAs are a subclass of miRNAs that localize to the nucleus and activate gene transcription through enhancer regions [124]. The oncogenic miR-492 functions as an NAmiRNA to modulate transcriptional programs in PC [112]. miR-492 activates enhancer loci within the genome, leading to upregulation of neighboring genes such as *NR2C1*, *NDUFA12*, and *TMCC3*. Through this mechanism, miR-492 promotes EMT and modulates the TGF-β signaling pathway, thereby enhancing PC cell proliferation, migration, and invasion. In contrast, miR-200c also operates via the NAmiRNA–enhancer pathway but exerts tumor-suppressive effects. miR-200c directly binds to enhancer regions and activates the transcription of adjacent genes, significantly inducing *PTPN6* expression, which in turn suppresses tumor cell proliferation [117].

These noncanonical miRNAs have not been extensively studied in PC, but in other diseases, they have been shown to possess noncanonical functions. For example, in non-small cell lung cancer, miR-744 was shown to interact with the c-Fos promoter and directly enhance c-Fos transcription [57]. Instead of repressing gene expression through the canonical 3′ UTR–mediated mechanism, this promoter-targeting activity activates AP-1–dependent oncogenic signaling and promotes cancer cell proliferation, migration, invasion, and tumor progression. In the context of gastric cancer, miR-558 binds directly to the promoter of *HPSE* within the nucleus, thereby disrupting the transcriptional repressive binding of *SMAD4* in an AGO1-dependent manner [115]. The process was demonstrated to activate *HPSE* transcription, consequently promoting tumor growth, invasion, metastasis, and angiogenesis. Another example is miR-215-5p, which is overexpressed in glioma and noncanonically regulates the tumor suppressor gene *PCDH9* [116]. miR-215-5p was observed to bind to the 3′ UTR of *PCDH9*, leading to mRNA degradation, and to directly interact with the *PCDH9* promoter, thereby repressing transcription. This dual regulatory mechanism was shown to markedly reduce *PCDH9* expression, resulting in enhanced proliferation, migration, and invasion of glioma cells, accompanied by suppressed apoptosis, and ultimately promoting tumor progression.

Taken together, these findings indicate that noncanonical miRNAs can regulate not only classical post-transcriptional targets but also transcriptional programs and the TME in PC. Although research on noncanonical miRNAs in PC remains limited, evidence from other cancer types suggests that they may similarly modulate tumor initiation, progression, metastasis, and drug resistance. Therefore, further investigation into their nuclear regulatory and epigenetic functions in PC is warranted, with potential implications for biomarker development and RNA or epigenetic-based therapeutic strategies.

## 4. Regulation of Long Non-Coding RNAs (lncRNAs) in PC

Another representative type of non-coding RNA is lncRNA, which is over 200 nucleotides in length [125]. Like mRNA, lncRNA is transcribed independently by RNA polymerase II in intergenic regions, generating pre-lncRNA, which undergoes 5′ capping and 3′ polyadenylation to form mature lncRNA [126]. The identification of lncRNAs was initially challenging, and their biological functions remained largely uncharacterized [126]. With the development of high-throughput RNA sequencing, a wide variety of lncRNAs have been identified, resulting in a rapid expansion of annotated lncRNAs in recent years [126]. Recent studies have demonstrated the diverse functions of lncRNAs and their critical roles in the regulation of gene expression, particularly in cancer [127,128]. lncRNAs interact with other RNA molecules, thereby modulating a variety of molecular mechanisms, including transcription, epigenetics, and post-transcriptional processes [129,130]. These interactions affect cancer-related cellular processes and have significant functional relevance in PC, highlighting their potential as therapeutic targets [130,131]. The function of lncRNAs is predominantly determined by their subcellular location [132,133]. Accordingly, this section focuses on representative oncogenic mechanisms of lncRNAs according to their nuclear or cytoplasmic localization and discusses how these mechanisms contribute to PC (Table 3).

### 4.1. Regulation of Cytoplasmic lncRNAs in PC

The lncRNAs located in the cytoplasm are related to translation regulation, with their most notable function being the process of miRNA sponging [152]. lncRNAs contain MREs, to which miRNAs bind, thereby allowing lncRNAs to act as sponges and inhibit miRNA function [153]. For instance, the lncRNA PART1 functions as an oncolncRNA by sponging miR-122, thereby promoting the malignant progression of PC [134]. Overexpression of PART1 is associated with increased tumor size, advanced T stage, and vascular invasion, as well as poor prognosis, and functionally promotes cell proliferation and invasion while inhibiting apoptosis. In the case of LINC01232, the lncRNA is upregulated in serum and tissues of PC and is associated with advanced TNM stage and poor prognosis, functioning as an oncogenic lncRNA. Bioinformatics analyses indicated that LINC01232 sponges miR-204-5p, miR-370-5p, and miR-654-3p, thereby promoting PC progression and worsening prognosis. LINC01232 has been suggested as a diagnostic and prognostic biomarker for PC based on its aberrant expression and clinical relevance [135]. In this manner, the function of lncRNAs is to sponge miRNAs, and because these miRNAs, as outlined in Section 3, inhibit mRNAs, the resultant lncRNA–miRNA–mRNA TRIAD forms a ceRNA network [154].

CeRNAs are composed of lncRNAs, miRNAs, and mRNAs, in which lncRNAs and mRNAs share the same miRNAs and compete for binding [155]. Subsequent studies have suggested that ceRNA networks provide a useful framework for understanding gene regulatory mechanisms [156]. Within this framework, the identification of specific lncRNA–miRNA–mRNA TRIADs has been proposed as a potential approach for exploring therapeutic strategies and biomarker targets in complex diseases [157,158]. In PC, TRIAD also increases the expression of oncogenes or inhibits TSGs, which activates cancer-related pathways such as cell growth, infiltration, and metastasis [159,160]. Related studies have recently increased in PC, where multi-target identification is important. For instance, in PC, aberrant upregulation of the lncRNA HOXA10-AS sponged miR-340-3p, thereby impairing the regulatory effect of miR-340-3p. As a result, expression of HTR1D, a direct target of miR-340-3p, is increased, and HTR1D promotes cancer cell proliferation and migration while inhibiting apoptosis through the PI3K–AKT pathway. Conversely, downregulation of HOXA10-AS leads to increased miR-340-3p levels and decreased HTR1D expression, suppressing PC progression in an AKT-dependent manner [136]. In the case of the dysregulated lncRNA ZFAS1 in PC, ZFAS1 sponged miR-497-5p, thereby sequestering miR-497-5p from its direct target HMGA2 and increasing HMGA2 expression. As a result, the expression of HMGA2-mediated downstream oncogenic programs is enhanced, promoting pancreatic cancer cell proliferation, migration, invasion, tumor growth, and metastasis [137]. Conversely, the TSlncRNA LINC01963 is downregulated in PC tissues and cell lines. LINC01963 sponged the oncomiR miR-641, relieving the repression of TMEFF2 by miR-641. Consequently, activation of the LINC01963/miR-641/TMEFF2 axis increases TMEFF2 expression, which reduces cell proliferation, migration, and invasion, induces cell cycle arrest, and promotes apoptosis, ultimately suppressing PC progression [144]. DGCR5 is another lncRNA that is downregulated in PC and plays a role in sponging miR-27a-3p [145]. Downregulation of miR-27a-3p induces activation of p38 MAPK and apoptosis in PC cells by regulating the expression of *BNIP3*.

Cytoplasmic lncRNAs also regulate cell signaling and gene expression, modulating mRNA stability and protein translation [161]. For example, lncRNA TSLNC8 binds with the oncogenic RNA-binding protein (RBP) HuR to form a complex that binds to the 3′ UTR of *CTNNB1*, thereby enhancing mRNA stability [138]. The stabilization of *CTNNB1* consequently increases β-catenin expression, activates the Wnt signaling pathway, and promotes cancer cell proliferation and invasion. In other words, TSLNC8 functions as a scaffold for HuR, stabilizing oncogenes and exerting tumor-promoting effects. Similarly, ST18-AS1 acts as a scaffold by interacting with the RBP FUS, which in turn increases the mRNA stability of the TSG *ST18*, thereby inhibiting cancer cell proliferation and promoting apoptosis in PC [146]. Consistently, elevated ST18-AS1 expression correlates with improved long-term survival in patients with PC, underscoring its promise as a prognostic biomarker. Taken together, cytoplasmic lncRNAs drive PC progression through lncRNA–miRNA–mRNA TRIAD-based regulatory networks, enabling coordinated and network-level control of gene expression at the post-transcriptional level.

### 4.2. Regulation of Nuclear-Localized lncRNAs in PC

Most nuclear-localized lncRNAs regulate gene expression at the transcriptional level [162]. These lncRNAs function as multifunctional regulators by interacting with chromatin, modulating transcriptional complexes, and controlling chromatin accessibility, thereby enabling precise regulation of gene expression [163,164]. Collectively, these mechanisms underscore the critical involvement of lncRNAs in cancer initiation and progression through the regulation of oncogene and TSG expression [165].

First, nuclear lncRNAs interact with chromatin to modulate gene-associated methylation and acetylation states [166]. This regulation is mediated by the recruitment of histone-modifying enzymes or chromatin regulatory proteins to specific genomic loci [167]. Consequently, the activation or repression of oncogenes and TSGs serves to regulate cancer-associated biological processes, including cell proliferation, survival, invasion, and metastasis. This regulatory process is initiated by the recruitment of histone-modifying complexes to specific genomic regions, resulting in alterations to histone marks and subsequent activation or repression of transcription [164,168]. For instance, the lncRNA PLACT1 has been observed to induce the expression of the heterochromatin protein hnRNPA1 at the *IκBα* promoter, thereby increasing H3K27me3 levels and repressing *IκBα* transcription [139]. As a result, NF-κB signaling is sustained, promoting PC progression. Similarly, HOTAIR has been shown to associate with EZH2 to induce heterochromatin formation [140]. The resulting repression of tumor-suppressive miRNAs, such as miR-34a, contributes to accelerated PC cell growth.

Furthermore, certain lncRNAs have been observed to bind to RNA polymerase II or TFs, thereby modulating the initiation of transcription for specific cancer-related genes [169]. These lncRNAs regulate gene transcription by inducing TFs to the promoter region of TGs or by interrupting the binding of TFs. These regulatory mechanisms have a direct impact on key cancer-related pathways, including the cell cycle, cell division, and apoptosis [169]. For example, in the case of RUNX1-IT1, it has been shown to bind to the RUNX1 TF within the nucleus of PC cells, thereby recruiting *RUNX1* into the promoter region of the *c-FOS* gene and promoting *c-FOS* transcription [141]. This enhances cell proliferation, migration, and infiltration of PC and confirms that patients with higher RUNX1-IT1 expression have a poor prognosis. Similarly, in the case of CTD-3252C9.4, it has been shown to inhibit the *IFI6* gene through a transcription inhibition complex [148]. CTD-3252C9.4 interacts with the TF IRF1, thereby preventing IRF1 binding to the *IFI6* promoter and suppressing *IFI6* transcription. This regulatory mechanism consequently inhibits PC cell proliferation and promotes apoptotic cell death.

Collectively, nuclear lncRNAs exert control over gene expression by modulating chromatin states and transcriptional complexes, thereby governing key oncogenic and tumor-suppressive programs in PC. These regulatory functions position nuclear lncRNAs as promising biomarkers of disease progression and as potential therapeutic targets for disrupting aberrant gene regulatory networks in PC.

## 5. Conclusions

PC remains one of the most lethal malignancies, largely due to late diagnosis, aggressive biological behavior, and the limited therapeutic efficacy of current treatment strategies. Despite the identification of key driver PCGs, *KRAS*, *TP53*, *CDKN2A*, and *SMAD4*, through extensive genomic studies, therapeutic approaches targeting these genes alone have yielded only limited clinical benefit. This limitation is indicative of the inherent molecular complexity of PC, whereby tumor progression is governed not by isolated genetic alterations but by interconnected regulatory networks.

**Figure 1:**
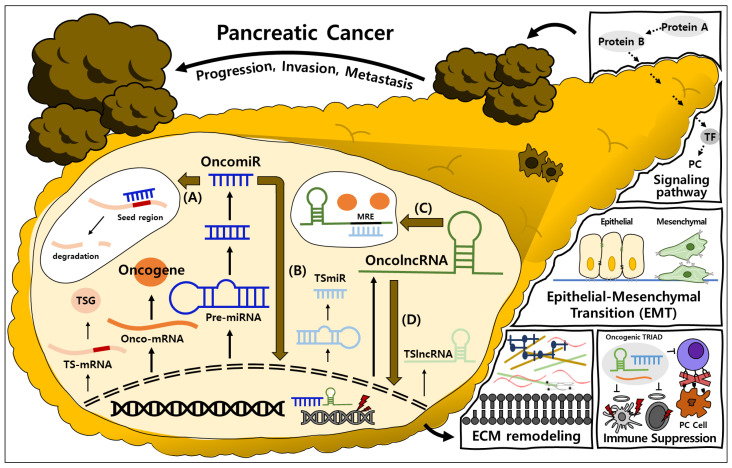
An overview of the biogenesis and regulatory mechanisms of the TRIAD that promote PC progression: (A) Canonical function of miRNAs. OncomiRs bind to the seed regions of TSG mRNAs, leading to mRNA degradation. (B) Noncanonical function of miRNAs. OncomiRs translocate into the nucleus and regulate transcription by activating oncogenes or repressing TSGs. (C) miRNA sponging by lncRNAs. OncolncRNAs contain MREs that competitively bind TSmiRs, thereby inhibiting their regulatory functions. (D) Nuclear functions of lncRNAs. OncolncRNAs localize to the nucleus and modulate transcriptional processes. PC: pancreatic cancer; miRNA: microRNA; oncomiR: oncogenic miRNA; TSG: tumor suppressor gene; mRNA: messenger RNA; lncRNA: long non-coding RNA; oncolncRNA: oncogenic lncRNA; MRE: miRNA response elements; TSmiR: tumor-suppressive miRNA.

This review provides a comprehensive overview of the regulatory landscape of PC, centering on PCGs, miRNAs, and lncRNAs, with particular emphasis on the lncRNA–miRNA–mRNA TRIAD as a key regulatory framework (Figure 1). Accumulating evidence indicates that effective multi-target strategies in PC require a comprehensive understanding of individual regulatory components, including PCGs, miRNAs, and lncRNAs. Each of these components independently governs key oncogenic processes such as aberrant signaling activation, TME remodeling, EMT, immune evasion, and therapeutic resistance. Building upon their individual regulatory roles, increasing attention has been directed towards the lncRNA–miRNA–mRNA TRIAD, which integrates these layers into a coordinated network. The TRIAD discussed throughout this review repeatedly influences shared signaling pathways, including Wnt, MAPK, and PI3K–AKT signaling. Across different studies, distinct TRIAD components modulate the same pathways, resulting in overlapping malignant phenotypes in PC. Shared regulation of identical pathways provides a basis for the limited clinical efficacy observed with previous single-target therapeutic strategies. Inhibition of a single regulator is suggested to be compensated for by alternative regulators functioning within the same pathway. In this context, multi-target approaches that simultaneously target multiple TRIADs within the same pathway may provide a more effective strategy for suppression and improved therapeutic efficacy in PC.

Therapeutic strategies targeting the TRIAD focus on the coordinated regulation of lncRNA, miRNA, and mRNA as a functional module, rather than on individual components. Single-target approaches often produce only temporary effects because compensatory pathways within the network can restore regulatory balance. The targeting of oncomiRNA within a TRIAD can affect multiple associated TSGs and TSlncRNAs concurrently, thereby increasing therapeutic impact. The modulation of two components together, such as miRNA and mRNA, miRNA and lncRNA, or mRNA and lncRNA, allows restoration of the balance of the ceRNA network, re-establishment of cellular homeostasis, and suppression of tumor progression. Network-based multi-target strategies provide a more effective approach than conventional therapies that target individual molecules. Furthermore, the rapid responsiveness and disease-specific expression patterns of ncRNAs suggest strong potential as diagnostic and prognostic biomarkers, especially in PC, where early detection remains challenging. Ultimately, continued investigation of ncRNA-centered regulatory networks will play a key role in overcoming therapeutic resistance and improving clinical outcomes in PC.

Nevertheless, several limitations remain in current ncRNA research in PC. Despite the growing recognition of TRIAD-mediated regulation, research in PC has remained disproportionately focused on PCGs, while ncRNAs, particularly lncRNAs, have been relatively underexplored. In silico resources for ncRNAs, particularly lncRNAs, remain limited, with the existing databases showing a marked bias towards a small number of well-characterized ncRNAs. Furthermore, the integration of molecular profiling data with clinical outcomes remains inadequate, largely due to the limited survival time of PC patients, which restricts the capacity for long-term observation of disease progression and therapeutic response. The restricted availability of PC tissues further restricts experimental validation and large-scale functional studies. In addition, more research, including studies on delivery systems, is needed to enable the application of ncRNAs as therapeutic agents or biomarkers. Collectively, these challenges underscore the necessity for future studies to address the existing gaps in ncRNA-centered PC research.

## Figures and Tables

**Table 1:** Classification of PC–related PCGs by gene type and function, with experimental validation.

Type	Category	PCGs	Impact on PC	Function	Interaction Element	Pathway	Clinical Value	Cell Line	Human Sample	Study Model	Ref
Oncogene	Pathway	*KRAS*	Tumor maintenance, progression	Diverts glucose metabolites into anabolic pathways to support growth	Glucose, Glutamine, *RPE*, *RPIA*	MAPK signaling pathway	Therapeutic target	Murine PC cell lines, human PDAC-derived cells	Human PDAC tissue sections	In vitro, in vivo	[45]
		*MYC*	Instructions and maintenance of the PDAC phenotype	Regulates global transcription and maintains cellular proliferation/identity	RNA Polymerase I & II, Max	Wnt signaling pathway	Therapeutic target	P493-6	Patient-derived tumor datasets	In vitro, in vivo	[46]
		*LETM2*	Progression, development	Accelerates proliferation, migration, and invasion	No interacting element	PI3K–AKT–mTOR signaling pathway	Diagnosis, Prognostic, Therapeutic	MIA PaCa-2, SW1990, BxPc-3	Adjacent normal 60, Tumor 60	In vitro, in vivo, in silico	[47]
		*UBE2C*	Progression	High expression of *UBE2C* is associated with poor overall survival. Inducing viability, migration, invasion, glucose uptake, and ATP production stabilizes EGFR and HGF	*EGFR*	PI3K–AKT signaling pathway	Prognosis	ANC1, Mia-Pada 2, HPDE6-C7	Adjacent normal 16, Tumor 16	In vitro, in silico	[48]
		*HIST3H2A*	Progression, Poor prognostic indicator	Modulates cell cycle progression and promotes abnormal proliferation	Chromatin, Histone modifiers	JAK–STAT signaling pathway	Diagnostic and prognostic Biomarker	BxPC-3, PANC-1, AsPC-1, SW1990	TCGA (The Cancer Genome Atlas) PDAC cohort	In silico	[49]
	TME	*YAP1*	Promotes progression, acts as an independent prognostic factor	Regulates transcriptional activation and ECM remodeling	*YAP1*, TEAD family, Collagen/ECM proteins	Hippo signaling pathway	Prognostic marker for overall survival	MIA PaCa-2, PANC-1, BxPC-3	Tumor 103	In vitro, in silico	[50]
		*MET*	Drives progression via TME modulation and immune evasion	Regulates immune cell infiltration and chemosensitivity	TME-related signature genes	Immune infiltration & TME remodeling	Risk score for prognosis and therapy response	N/A (Mainly Database-driven)	TCGA-PAAD, GEO datasets	In silico	[51]
		*DCLK1*	Promotes PC progression and metastasis via m6A regulation	*ALKBH5* stabilizes *KIF3C* mRNA; induces viability and migration	*ALKBH5*, *KIF3C* (m6A-dependent)	m6A-mediated mRNA stability	Therapeutic target, prognostic biomarker	BxPC-3, PANC-1, MIA PaCa-2	Adjacent normal, Tumor	In vitro, in vivo	[52]
		*TWIST*	Facilitates proliferation, metastasis, and immune evasion	Twist1 induces *VISTA* expression, promotes EMT, and T cell suppression	*TWIST1*, *VISTA*, CD8+ T cells	EMT and Immune Checkpoint Signaling	Target for combined immunotherapy	Mia PaCa-2, AsPC-1, CAPAN-1	TCGA datasets and validated clinical samples	In vitro, in silico	[53]
		*PADI1*	Unknown impact on PC	promote cell migration, invasion, and the epithelial–mesenchymal transition (EMT)	No interacting element	ERK1/2-p38 signaling	Treatment	CFPAC-1, PANC-1, PL45, Bxpc-3, HPAC, and hTERT-HPNE	No human sample data	In vitro, in silico	[54]
TSG	pathway	*HNF1A*	Enhance growth, metabolic shift	Regulates glucose metabolism and survival	*MUC4*, *HER2*	Akt/mTOR	Metabolic therapeutic target	CD18/HPAF, CAPAN-1	PDAC patient tissues	In vitro, in vivo	[55]
		*SOX15*	Loss of *GATA6* drives EMT, Progression	Maintains epithelial state, suppresses the basal-like subtype	*GATA6*, *OCT4*, *SOX2*	Wnt/β-catenin signaling pathway	Subtype stratification (Classical vs. Basal)	BxPC-3, PANC-1	Patient-derived xenografts (PDX)	In vitro, in vivo, in silico	[56]
		*SMAD4*	Regulates metastatic potential and TME	Mediates tumor suppression vs. metastasis	*SMAD4*, TGF-β receptors	TGF-β pathway	Prognostic marker for metastasis	MIA PaCa-2, AsPC-1	Metastatic PDAC patient cohorts	In vivo, in vitro	[57]
		*PTEN*	Promotes tumor cell invasion and growth	Regulates cytoskeletal dynamics and signaling	*MYO1B*, Actin	PI3K–AKT	Therapeutic target	MIA PaCa-2, PANC-1	PC patient tumor samples	In vitro, in vivo	[58]
		*DUSP6*	Primary driver of tumor initiation	Loss of cell cycle control (G1/S checkpoint)	p16, CDK4/6, Rb	MAPK	Diagnostic marker for familial/sporadic PC	Various primary PDAC cultures	Primary pancreatic tumor biopsies	In vitro, in silico	[59]
	TME	*ARID1A*	Tumor formation, tumorigenesis	Transcriptional repressor of FASN	*A* *RID* *1* *A*	No related pathway	NA	ASPC-1, MIAPaCa-2, BxPC-3	KAR mouse tissues	In vivo, in vitro, in silico	[60]
		*PCDH17*	Metastasis, progression, development, proliferation, invasion	Unknown function	No interacting element	No related pathway	Therapeutic target	NA	Adjacent normal 3, Tumor 6	In silico, ex vivo	[61]
		*CEBPA*	Carcinogenesis	Clonogenic growth inhibition	*E2F1*	No related pathway	Therapeutic target	PANC-1, AsPC-1, BxPC3, Maraca, Capan-2, HPAC, and HPAF-II	Normal 10, Tumor 32	In vitro	[62]
		*KLF4*	Progression	Inhibits EMT and metastasis	CAV1	KLF4/Cav-1 signaling pathway	Diagnostic and therapeutic target	AsPC-1	Normal 10, Tumor 70	In vitro, in vivo, in silico	[63]
		*DAB2*	Development, metastasis, progression	Promotion of EMT and CSC marker expression with enhancement of TGFβ-induced EMT, Maintenance of the epithelial phenotype by Dab2	*TGFBR1*, *TGFBR2*	TGFβ signaling pathway, MAPK/ERK signaling pathway	Prognostic	COLO357, BxPC3, AsPC1, Panc1, MiaPaCa2	Normal 5, Tumor 18	In vitro, in silico	[64]

**Table 2:** Classification of PC–related miRNAs by miRNA type and function, with experimental validation.

Type	Category	miRNAs	Impact on PC	Function	Interaction Element	Pathway	Clinical Value	Cell Line	Human Sample	Study Model	Ref
OncomiR	Canonical	miR-491-5p	Progression, development	Unknown function	*miRTP53*	*TP53* signaling pathway	Prognostic biomarker	No cell line	PC tissue (n = 179)/normal tissue (n = 171)	In silico	[110]
		miR-155	Unknown impact in PC	Promote reactive oxygen species (ROS) production, enhance cell proliferation, promote cell transformation, and promote tumor growth	*F* *OX* *O3*	MAPK/ERK signaling pathway NF-κB signaling pathway	Unknown	T-Rex/K-Ras, Capan-2, AsPC-1, PANC-1, BxPC-3, HPDE, HPNE	Pancreatic ductal carcinoma tissues (81 cases) paired non-neoplastic pancreatic tissues	In vitro, in vivo	[102]
		miR-642a-5p	Development, progression, tumorigenesis, metastasis, carcinogenesis	Inhibits growth, migration, and invasion, promotes apoptosis	*KRT19*	Wnt/b-catenin pathway	Prognostic indicator, predictive marker, potential target	BxPC-3, AsPC-1, SW 1990, PANC-1, HPDE6-C7, HEK293T	53 pairs of PC and matched paracancerous tissues	In vitro, in vivo, in silico	[103]
		miR-194-5p	Tumor progression, tumor growth, malignant process, migration, invasion, proliferation, EMT process	Inhibits the migration, invasion, proliferation, and EMT process of PC cells	*PD-L1*	*PD-1/PD-L1* pathway	Prognostic biomarker therapeutic target	Panc1, Panc02, HEK-293T	No human sample data	In vitro, in vivo in silico	[104]
		miR-122-5p	Tumor-suppressive tumor suppressor	Restrain PC cell proliferation, accelerate apoptosis, decrease glutamine consumption	*ASCT2*	No related pathway	Poor prognosis	No cell line	No human sample data	In vitro ex vivo	[111]
	Noncanonical	miR-492	Progression	Promote EMT, viability proliferation, migration, invasion	*NR2C1/NDUFA12/TMCC3*	*NR2C1*-TGF-β/Smad3 pathway	Diagnostic and therapeutic target	CAPAN1, CAPAN-2, NOPR1, CFPAC-1, SUIT2, SW1990, PANC-1, MiaPaCa-2, BxPC-3, AsPC-1, and KP3	54 PC tissues and paracancerous tissues	In vitro, in vivo, in silico	[112]
		miR-21	Tumorigenesis, tumor progression, carcinogenesis, migration, invasion, proliferation	Proliferation, migration, invasion, tumorigenesis	NF-κB and AP-1 TF binding sites	NF-κB signaling pathway Rho GTPase signaling pathway	Prognostic biomarker poor prognosis	PANC-1, AsPC-1, BxPC-3, CFPAC-1	No human sample data	in vitro in vivo	[113]
		miR-744	Unknown impact in PC	Binds to miRNA promoter in NSCLC	*FOS*	*AP-1*	Prognostic, prognostic marker, independent unfavorable prognostic marker	A549, H520, H1299, SPC-A-1, HBE	NSCLC tissues, adjacent noncancerous tissues: 15 matched pairs NSCLC tissue chips: 87 cases TCGA primary NSCLC: 456 cases	in vitro in vivo in silico	[114]
		miR-558	Unknown impact in PC	Binds to miRNA promoter in lung cancer	*HPSE*	No related pathway	Prognosis, survival, therapeutic targets	AGS, SGC-7901, MKN-45, MKN-28, HPSEC, GES-1, HUVEC	Fresh tumor and adjacent normal gastric specimens: n = 90 Paraffin-embedded sections: n = 50	In vitro in vivo in silico	[115]
		miR-215-5p	Unknown impact in PC	Binds to both the PCDH9 promoter and 3′UTR	*PCDH9*	No related pathway	Prognostic factor, therapeutic target	U251, U87, HEK293	Primary glioma tissues: n = 30 Normal brain tissues: n = 8	In vitro, in silico, ex vivo	[116]
TSmiR	Canonical	miR-217	Development, progression	Inhibit tumor cell growth, inhibit anchorage-independent colony formation, decrease xenograft tumor growth	*KRAS*	RAS/PI3K/AKT signaling pathway	Therapeutic agent, miRNA-based PDAC therapy	PANC-1, MIAPaCa-2, AsPC-1, BxPC-3, P1, P3, P7	PDAC tissues: 21, paired adjacent normal pancreas: 21	In vitro, in vivo, in silico	[105]
		miR-30d	Development, progression, tumorigenesis, carcinogenesis, metastasis	Inhibit proliferation, induce apoptosis, induce G1/S cell cycle arrest, suppress migration, suppress invasion, inhibit growth, suppress metastasis	*SOX4*	PI3K-AKT signaling pathway	Prognostic biomarker, diagnostic markers, therapeutic target	BxPC-3, Capan-2, Mia PaCa-2, Panc-1, SW-1990, HPDE	PC tissues: 80 pairs, matched adjacent non-tumor tissues: 35 pairs	In vitro, in vivo, in silico	[106]
		miR-202	Tumorigenesis	Reduce proliferation, tumor volume, tumor weight, and glycolysis	*HK2*	No related pathway	Therapeutic	AsPC-1, BxPC-3, SW1990, PANC-1, HS766t, and hTERT-HPNE	101 PCs patients	In vitro, in vivo, in silico	[107]
		miR-128-3p	Pathogenesis	Inhibit EMT, invasion, and migration	*ZEB1*	No related pathway	Therapeutic	HPDE6c7, AsPC-1, BxPC-3, CFPAC-1, and PANC-1	No human sample data	In vitro, in silico	[108]
		mir-190b	Progression	Suppress cell proliferation, invasion, metastasis	*MEF2C* and *TCF4*	Wnt signaling pathway	Diagnostic biomarker and therapeutic target	AsPC-1, BxPC-3, HPC-Y5, SW1990, Capan-2, PANC-1 and MIA PaCa-2, normal human pancreatic ductal epithelial cell line HPDE	50 PDAC tissues, matched non-tumor adjacent tissue	In vitro, in vivo, in silico	[109]
	Noncanonical	miR-200c	Progression, metastasis, tumor growth, migration	NAmiRNA-enhancer	*PTPN6*	STAT3 signaling pathway	Therapeutic, prognostic	BxPC-3, MIA PaCa-2, PANC-1, AsPC-1, HEK293T	PDAC tissue: 96	In vitro, in vivo, in silico	[117]
		miR-24-1	Progression, metastasis, tumor growth, Warburg effect	NAmiRNA-enhancer	*FBP1*	No related pathway	Prognostic biomarker, therapeutic target	786-O, ACHN, HEK293T	RCC tissue and adjacent normal tissue, 42 paired samples	In vitro, in vivo, in silico	[118]
		miR-339	Unknown impact in PC	Inhibit invasion, and migration	*GPER1*	No related pathway	Treatment, prognosis, biomarker, therapeutic target	T47D, LM2-4175, HEK293T, MCF-10A	Breast cancer & adjacent normal (157 pairs), ovarian tissues (23 cancer, 6 normal), endometrial tissues (10 cancer, 3 normal)	In vitro, in vivo, in silico	[119]
		miR-126-5p	Unknown impact in PC	Suppress atherosclerosis	*CASP* *3*	mTOR signaling pathway	Therapeutic relevance, diagnostic marker, surrogate marker, prognostic factor	HUVECs	Human carotid artery specimens: n = 7	In vitro, in vivo, in silico	[120]
		miR-26A1	Tumorigenesis, proliferation, metastasis, malignant behavior, migration	Inhibit invasion, and migration in NSCLC	*VILL*	No related pathway	Therapeutic target, biomarker	A549, H1703, HEK293T, MRC5, BEAS-2B	NSCLC tissue and adjacent lung tissue: 11 pairs, LUAD 7, LUSC 4	In vitro, in silico	[121]

**Table 3:** Classification of PC–related lncRNAs by lncRNA type and function, with experimental validation.

Type	Category	lncRNAs	Impact on PC	Function	Interaction Element	Pathway	Clinical Value	Cell Line	Human Sample	Study Model	Ref
Onco-lncRNAs	Plasmic	PART1	Progression	Promote proliferation, invasion, and suppress apoptosis	miR-122	No related pathway	Therapy	AsPC-1, Panc-1, SW1990, BxPC-3, normal human pancreatic ductal epithelial line (HPDE6c7	Forty-five samples of fresh cancer tissues and paratumor normal tissues	In vitro, in silico	[134]
		LINC01232	Unknown impact in PC	Unknown function	miR-204-5p, miR-370-5p, and miR-654-3p	No related pathway	Diagnosis and prognosis	No cell line	A total of 108 patients’ tumors and normal tissues and serum. 60 healthy serums	In silico	[135]
		HOXA10-AS	Progression	Promote proliferation, migration	miR-340-3p	AKT signaling pathway	Diagnosis and treatment	BxPC-3, SW1990, PANC-1, CFPAC-1, AsPC-1, T3M4, and HPDE6-C7	4 random pairs among 90 pairs of PC and paracancerous tissue microarrays	In vitro, in vivo, in silico	[136]
					*HTR1D*						
		ZFAS1	Progression	Promote proliferation, migration and metastasis	miR-497-5p *HMGA2*	No related pathway	Diagnosis and treatment	BxPC-3, SW1990, AsPC-1, PANC-1, and HPNE	PC tissues and adjacent normal tissues	In vitro, in vivo, in silico	[137]
		TSLNC8	Pathogenesis	Enhance proliferation and metastasis, invasion	*ELAVL1*	Wnt signaling pathway	Unknown	HPDE, AsPC-1, Capan-2, SW1990, PANC-1, PaCa-2, and BxPC-3	Seventy patients with PC and adjacent normal tissues	In vitro, in vivo, in silico	[138]
	Nucleic	PLACT1	Progression and tumorigenesis	Promote proliferation, migration, and invasion	*HN* *RNPA1*	NF-κB signaling	Therapeutic	AsPC-1, BxPC-3, Capan-2, CFPAC-1, MIA PaCa-2, PANC-1, SW1990, and HPNE	166 paired primary PDAC	In vitro, in vivo, in silico	[139]
		HOTAIR	Tumorigenesis	Promote proliferation and tumor volume	*EZH2*	No related pathway	Therapy	BxPC3, CAPAN2, CFPAC1, PANC04.03, PANC1, SW1990, HEK293T, and HPDE	No human sample data	In vitro, in vivo	[140]
		RUNX1-IT1	Progression	Promote proliferation, migration, and invasion	*RUNX1 TF*	No related pathway	Prognostic biomarker, therapeutic	AsPC-1, BxPC-3, CFPAC-1, PANC-1 and SW1990	Eighty-three freshly frozen PC samples (38 with adjacent noncancerous tissues), 16 normal pancreatic tissue samples, and 175 PC tissues for tissue microarrays	In vitro, in vivo, in silico	[141]
		SLC7A11-AS1	Tumorigenesis, progression, metastasis	Reduce intracellular ROS, maintain cancer stemness, potentiate sensitivity toward gemcitabine; block NRF2 ubiquitination; prevent proteasomal degradation of nuclear NRF2	*B* *TRC1*	No related pathway	Therapeutic target, poor prognosis	BxPC-3, BxPC-3-Gem, PANC-1, AsPC-1, CFPAC-1, 293T	PDAC tumor & adjacent normal tissues (n = 27 pairs)	In vitro, in vivo	[142]
		LINC01614	Tumorigenesis, tumor progression, tumor growth, proliferation, migration, invasion, epithelial–mesenchymal transition	Promote cell proliferation, promote migration and invasion, induce epithelial–mesenchymal transition, stabilize β-catenin protein, activate WNT/β-catenin signaling	*CTNNB1*	Wnt/β-catenin signaling pathway	Prognostic (disease-free survival)	Panc-1, SW1990, BxPC-3, MIA-PaCa, HPDE	PC tissue 20, adjacent non-tumor pancreatic tissue 20	In vitro, in vivo	[143]
TS-lncRNAs	Cytoplasmic	LINC01963	Progression	Suppress proliferation, invasion, and migration, and promote apoptosis and cell cycle arrest	miR-641	No related pathway	Treatment	PANC-1, CFPAC-1, BxPC-3, SW1990, AsPC1, and HPDE6-C7	67 PC patients	In vitro, in vivo, in silico	[144]
					*TMEFF2*						
		DGCR5	Progression and development	Promote apoptosis and inhibit tumor growth	miR-27a-3p	p38 MAPK pathway	Treatment and prognosis	SW1990, PANC-1, and HPDE6-C7	PaCa tissues and adjacent normal tissues (n = 20)	In vitro, in vivo, in silico	[145]
					*BNIP3*						
		ST18-AS1	Progression	Inhibit proliferation and promote apoptosis	*FUS*	No related pathway	Therapeutic	BxPC-3, MIA Paca-2, CFPAC-1, SW1990, ASPC-1, PANC-1	55 tumors and 26 non-tumorous adjacent tissues	In vitro, in vivo, in silico	[146]
		LINC00261	Progression, migration, invasion, chemoresistance	Suppressed glycolysis and proliferation; induced cell cycle arrest and apoptosis; reduced c-myc expression; reduced c-myc mRNA stability	miR-222-3p	HIPK2/ERK/c-myc pathway	Prognostic marker, targeted therapy	Aspc1, Bxpc3, Capan1, Mia-PaCa2, Panc1, Sw1990, Patu8988, HPNE, HEK-293T	PC tissues, adjacent normal tissues (87 pairs)	In vitro, in vivo, in silico	[147]
	Nuclear-localized	CTD-3252C9.4	Progression	Promote apoptosis and suppress migration, invasion, and proliferation	IRF1 TF	No related pathway	Therapeutic	Panc-1, MIA Paca- 2, SW1990, BXpc-3, HPDE	20 pairs of human pancreatic tissues and adjacent normal	In vitro, in vivo, in silico	[148]
		LINC01197	tumor growth, tumor progression	Inhibit PC cell proliferation, suppress β-catenin–dependent transcription	*CTNNB1*	Wnt/β-catenin signaling pathway	Prognostic biomarker	PANC-1, BxPC-3, AsPC-1, CFPAC-1	pancreatic ductal adenocarcinoma tissues and adjacent normal tissues (80 pairs)	In vitro, in vivo	[149]
		MEG3	development, metastasis, vascular invasion	Inhibit cell proliferation, induce apoptosis, induce G1 phase arrest, inhibit cell invasion, inhibit cell migration	No interacting element	PI3K/AKT/Bcl-2/Bax/Cyclin D1/P53 signaling pathway, PI3K/AKT/MMP-2/MMP-9 signaling pathway	Unknown	PANC-1	PC tissue (n = 30), carcinoma adjacent tissue (n = 30)	In vitro, ex vivo	[150]
		LINC00173	tumorigenesis, tumor development, cancer progression, initiation and progression, metastasis, angiogenesis, chemoresistance, tumor growth, invasion, migration, proliferation, apoptosis, cell cycle regulation	Promote proliferation, migration, and invasion, enhance tumorigenesis and angiogenesis, promote chemoresistance, regulate glucose metabolism, inhibit apoptosis, suppress proliferation and invasion, promote apoptosis, inhibit tumor growth	SPHK1	NF-κB signaling pathway, Akt/NF-κB signaling pathway, glucose metabolism pathway, pentose phosphate pathway, glycolysis	Diagnostic biomarker, prognostic biomarker, therapeutic target, chemotherapeutic sensitizer	MIA PaCa-2, MDA-MB-231, TK6, HQ-MT	Colorectal cancer tissue (57 paired samples)	In vitro, in vivo, in silico	[151]
		STXBP5-AS1	chemoresistance, metastasis, stem cell-like properties, tumor incidence, tumorigenic capacity	Inhibit chemoresistance, increase Gemcitabine sensitivity, inhibit invasion, suppress lung metastasis, induce apoptosis, suppress sphere formation, recruit EZH2 to ADGB promoter, increase ADGB promoter DNA methylation, and epigenetically inhibit ADGB transcription	*EZH2*	No related pathway	Prognostic	AsPC-1, SW1990, Capan-2, CFPAC-1, PANC-1, Mia PaCa-2, hTERT-HPNE, PANC-1/GR, Mia PaCa-2/GR	PC tumor 60, paired adjacent normal 60	In vitro, in vivo	[122]

## Data Availability

No new data were created or analyzed in this study. Data sharing is not applicable to this article.

## Abbreviations

The following abbreviations are used in this manuscript:

ceRNA	Competing Endogenous RNA
ECM	Extracellular Matrix
EMT	Epithelial–Mesenchymal Transition
lncRNA	Long Non-Coding RNA
MAPK	Mitogen-Activated Protein Kinase
miRNA	MicroRNA
MRE	miRNA Response Element
mRNA	Messenger RNA
NAmiRNA	Nuclear Activating miRNA
ncRNA	Non-Coding RNA
OncomiR	Oncogenic miRNA
PC	Pancreatic Cancer
PCG	Protein-Coding Gene
RBP	RNA-Binding Protein
ROS	Reactive Oxygen Species
TCGA	The Cancer Genome Atlas
TF	Transcription Factor
TG	Target Gene
TME	Tumor Microenvironment
TSG	Tumor Suppressor Gene
TSmiR	Tumor-Suppressive miRNA
UTR	Untranslated Region
